# Comparison of different commercial kits for HER2 testing in breast cancer: looking for the accurate cutoff for amplification

**DOI:** 10.1186/bcr1770

**Published:** 2007-10-01

**Authors:** Anne Cayre, Florence Mishellany, Nicole Lagarde, Frédérique Penault-Llorca

**Affiliations:** 1 Département de Pathologie, Centre Jean Perrin, 58 Rue Montalembert, BP392, 63011 Clermont-Ferrand Cédex, France; 2 Inserm UMR 484, Rue Montalembert, BP184, 63005 Clermont-Ferrand, France; 3 Institut Universitaire de Technologie, Ensemble Universitaire des Cézeaux, BP 86, 63170 Aubière, France; 4 CHU Hôpital Morvan, 5 Avenue Foch, 29609 Brest Cédex, France; 5 Université d'Auvergne, Faculté de Médecine, 28 Place Henri Dunant, BP 38, 63000 Clermont-Ferrand, France

## Abstract

**Introduction:**

Accurate determination of human epidermal growth factor receptor 2 (HER2) status is essential for optimal patient management with trastuzumab (Herceptin). However, standard guidelines do not specify a particular commercial kit, antibody or probe for testing, and discrepancies arise from variability between kits. The aim of this study was to compare the accuracy of four commercially available fluorescence/chromogenic *in situ *hybridisation (FISH/CISH) kits and validate one for the resolution of borderline immunohistochemistry (IHC) cases. The interpretation pitfalls, optimal threshold values, assay duration and complexity of each kit were also considered.

**Methods:**

The Food and Drug Administration (FDA)-approved dual-probe FISH assay PathVysion was chosen as the 'gold standard' against which pharmDx (dual-probe) and INFORM (mono-probe) FDA-approved FISH kits and the SPoT-Light CISH kit were compared. Tumours were also evaluated by IHC with the FDA-approved HercepTest kit and a validated in-house IHC protocol. Fifty-five patients with invasive breast carcinoma were selected as a representative proportion of HER2 IHC 2+ cases.

**Results:**

HER2 amplification was observed in 31% of tumours by PathVysion compared with 33% with pharmDx. The number of amplified tumours detected by INFORM and CISH varied with the threshold applied. Agreement was excellent between PathVysion and pharmDx (100%), good with SPoT-Light (89%; cutoff at least five signals per nucleus) and moderate with INFORM (76%; cutoff more than four signals per nucleus). Agreement with INFORM improved to 98% with a cutoff of at least six signals per nucleus.

**Conclusion:**

With an appropriate cutoff, the INFORM kit was comparable to dual-probe FISH kits for evaluating HER2 status. We validate and recommend CISH as an appropriate assay for HER2 scoring that is easy to interpret and requires equipment readily found in, or that can be adapted to, all pathology laboratories. For borderline IHC cases, dual-probe FISH analysis remains the most useful protocol to apply.

## Introduction

About 20 to 30% of breast cancer tumours are positive for amplification and overexpression of human epidermal growth factor receptor 2 (HER2), which is associated with poor prognosis and clinical outcome [1-3]. HER2 is targeted therapeutically by the humanised monoclonal antibody trastuzumab (Herceptin; F Hoffman-La Roche Ltd, Basel, Switzerland).

Trastuzumab is effective only in patients whose tumours are positive for *HER2 *gene amplification and/or protein overexpression [4-7]. Therefore, accurate HER2 testing is essential for selecting patients eligible for trastuzumab-based therapies. Originally, HER2 status was determined by Southern blotting [3,8], but the present methods of evaluation combine immunohistochemistry (IHC) and fluorescence/chromogenic *in situ *hybridisation (FISH/CISH). Inconsistencies arise in HER2 scoring because of the variety of commercial assays available, which use different antibodies and probes. Although the College of American Pathologists [9] and a UK-based study [10] recommend IHC and FISH for HER2 testing, neither specify a particular commercial kit.

In the present study, the Food and Drug Administration (FDA)-approved dual-probe FISH assay PathVysion (Abbott France SAS, Rungis, France) was used as 'gold standard' against which the FDA-approved dual-probe pharmDx (Dako France SAS, Trappes, France) and mono-probe INFORM (Ventana Medical Systems SA, Illkirch, France) FISH assays, plus the CISH assay SPoT-Light (Zymed Laboratories Inc., San Francisco, CA, USA) were compared. Invasive tumour specimens from 55 patients were evaluated for HER2 status with these different methods. This study is the first, to our knowledge, that compares three commercially available FISH analysis kits with CISH, and evaluates the respective thresholds of each assay.

## Materials and methods

### Samples

Tumour samples from 55 patients with invasive breast carcinoma were collected for evaluation between January 2001 and April 2003. All samples were fixed in neutral-buffered formalin and embedded in paraffin. Cases were selected to ensure a representative proportion of HER2 IHC 2+ cases, and blind assessments were made of the invasive tumour component of all samples by two independent pathologists.

The study was conducted in line with the Declaration of Helsinki and Good Clinical Practice, and with the approval of an independent Institutional Review Board.

### Immunohistochemistry

Tumour samples were assessed for HER2 status with the FDA-approved HercepTest kit (Dako) and a standardised in-house laboratory technique with the rabbit anti-human HER2 polyclonal A0485 (1:400 dilution; Dako) for 32 minutes [11]. Tissue sections (3 μm) were deparaffinised and rehydrated, and antigens were retrieved for 40 minutes in citrate buffer (pH 6.1) at 95°C. IHC was performed with the NexES automated immunostaining system and diaminobenzidine detection kit (Ventana Medical Systems SA). Staining intensity was graded in accordance with the HercepTest protocol system as 0, 1+, 2+ or 3+. Samples scored as 0 or 1+ were considered negative for HER2 overexpression, 2+ was weak positive and 3+ was strong positive, with complete membrane staining of more than 10% of tumour cells.

### Fluorescence *in situ *hybridisation

The FDA-approved PathVysion and pharmDx assays were used in accordance with the manufacturers' recommended protocols but with some minor modifications. The DNA probes, HER2-specific sequence probe (LSI HER2/neu), chromosome enumeration probe 17 (CEP17) and tissue sections were denatured for 5 minutes at 85°C (PathVysion) or 82°C (pharmDx) with a HYBrite instrument (Abbott France SAS). An additional wash in distilled water was performed before counterstaining and mounting with a solution of 4,6-diamidino-2-phenylindole (DAPI). As the intensity of fluorescence observed with the DAPI solution provided in the pharmDx kit was poor, the commercially available VECTASHIELD plus DAPI mounting medium (AbCys SA, Paris, France) was used instead for these samples. The results are reported as the ratio of average HER2:CEP17 signals per nucleus. Signal ratios of less than two were classified as non-amplified (NA), and ratios of two or more as amplified.

The INFORM assay, an automated system using the BenchMark automated staining system (Ventana Medical Systems SA), was used in accordance with the manufacturer's recommended protocols. Amplification was defined as more than four signals per nucleus, as stated in the manufacturer's guidelines. Additional thresholds were applied (more than five and at least six signals per nucleus) for comparison and optimisation. At least 30 nuclei were counted for signals in each section.

### Chromogenic *in situ *hybridisation

CISH analysis of HER2 amplification was performed with the SPoT-Light assay in accordance with the manufacturer's protocol. The DNA probe (HER2 SPoT-Light) and sections were denatured at 94 to 95°C and hybridised overnight at 37°C with a HYBrite instrument. Amplification was defined with two thresholds – more than five or at least six signals per nucleus – detected in more than 50% of nuclei or when a large signal cluster was detected. At least 30 nuclei were counted per section.

### Statistics

PathVysion was used as the 'gold standard' against which all protocols were compared, and agreement was quantified with pairwise kappa (κ) statistics. The analytical performance of these kits was also estimated for specificity and sensitivity. The pairwise χ^2 ^test was used to assess overvaluation or undervaluation of HER2 status.

## Results

HER2 status for all samples as determined by IHC, FISH and CISH are detailed in Table 1.

**Table 1:** Evaluation of HER2 status by IHC, FISH and CISH

Case ID	PathVysion	pharmDx	INFORM			CISH		In-house	HercepTest
			>4	>5	≥6	>5	≥6		
1	A	A	A	A	A	A	A	3	3
2	A	A	A	A	A	A	A	3	3
3	A	A	A	A	A	A	A	3	3
4	A	A	A	A	A	A	A	3	3
5	A	A	A	A	A	A	A	3	3
6	A	A	A	A	A	A	A	3	3
7	A	A	A	A	A	A	A	3	3
8	A	A	A	A	A	A	A	3	3
9	A	A	A	A	A	A	A	3	3
10	A	A	A	A	A	A	A	3	3
11	A	A	A	A	A	A	A	3	3
12	A	A	A	A	A	A	A	3	3
13	A	A	A	A	A	A	A	3	3
14	A	A	A	A	A	A	A	3	3
15	A	A	A	A	A	A	A	3	3
16	A	A	A	A	A	A	A	3	3
17	A	A	A	A	A	A	A	2	3
18	NA	NA	NA	NA	NA	NA	NA	2	3
19	NA	NA	NA	NA	NA	NA	NA	2	2
20	NA	NA	A	NA	NA	A	A	3	3
21	NA	NA	A	A	NA	A	NA	3	3
22	-	A	NA	NA	NA	NA	NA	2	3
23	-	NA	NA	NA	NA	NA	NA	2	1
24	NA	NA	A	NA	NA	A	A	1	3
25	NA	NA	A	A	A	A	NA	2	3
26	NA	NA	A	A	NA	A	A	2	2
27	NA	NA	A	NA	NA	NA	NA	2	2
28	NA	NA	A	A	NA	NA	NA	2	2
29	NA	NA	A	NA	NA	NA	NA	2	1
30	NA	NA	A	A	NA	NA	NA	1	2
31	NA	NA	NA	NA	NA	NA	NA	1	1
32	NA	NA	A	NA	NA	NA	NA	0	0
33	NA	NA	A	NA	NA	A	NA	0	1
34	NA	NA	A	NA	NA	NA	NA	0	2
35	NA	NA	A	NA	NA	NA	NA	0	0
36	NA	NA	NA	NA	NA	NA	NA	0	1
37	NA	NA	NA	NA	NA	NA	NA	0	1
38	NA	NA	NA	NA	NA	NA	NA	2	3
39	NA	NA	NA	NA	NA	NA	NA	2	3
40	NA	NA	NA	NA	NA	NA	NA	2	2
41	NA	NA	NA	NA	NA	NA	NA	2	2
42	NA	NA	NA	NA	NA	NA	NA	2	2
43	NA	NA	NA	NA	NA	NA	NA	2	1
44	NA	NA	NA	NA	NA	NA	NA	2	0
45	NA	NA	NA	NA	NA	NA	NA	0	2
46	NA	NA	NA	NA	NA	NA	NA	0	2
47	NA	NA	NA	NA	NA	NA	NA	0	2
48	NA	NA	NA	NA	NA	NA	NA	0	1
49	NA	NA	NA	NA	NA	NA	NA	0	1
50	NA	NA	NA	NA	NA	NA	NA	0	1
51	NA	NA	NA	NA	NA	NA	NA	0	0
52	NA	NA	NA	NA	NA	NA	NA	0	0
53	NA	NA	NA	NA	NA	NA	NA	0	0
54	NA	NA	NA	NA	NA	NA	NA	0	0
55	NA	NA	NA	NA	NA	NA	NA	0	0

HER2, human epidermal growth factor receptor 2; A, amplified; NA, non-amplified; IHC, immunohistochemistry; FISH, fluorescence *in situ *hybridisation; CISH, chromogenic *in situ *hybridisation.

### Immunohistochemistry

With the A0485 antibody-based in-house IHC protocol, 18 tumours (33%) were scored as IHC 3+, 17 (31%) as IHC 2+ and 20 (36%) as IHC 0/1+ (HER2 negative). In contrast, HercepTest identified 25 tumours (45%) as IHC 3+ and 12 (22%) as 2+ (HER2-positive); 18 (33%) were scored as IHC 0/1+ (HER2-negative). The κ coefficient between the two tests was 0.427 ± 0.083 (SD) and agreement was 58% if in-house IHC was considered as the reference standard. Concordance between the two protocols was 70% for HER2-negative IHC 0/1+ cases, 47% for IHC 2+ and 100% for HER2-positive IHC 3+ cases, if in-house IHC was considered as the reference standard. All IHC 3+ cases demonstrated complete membrane staining in at least 30% of tumour cells.

### Fluorescence *in situ *hybridisation

Two cases could not be evaluated with the PathVysion kit but were assessed and graded successfully using pharmDx and INFORM. PathVysion identified 17 tumours (31%) as amplified, in comparison with 18 (33%) by pharmDx. INFORM had three thresholds applied to determine the optimal cutoff for evaluating positive HER2 amplification. These thresholds were more than four signals per nucleus as recommended by the manufacturer, more than five signals per nucleus as recommended for CISH analysis, and at least six signals per nucleus as recommended by Zymed for the SPoT-Light CISH kit used in this study. Using a cutoff of more than four signals per nucleus, 30 tumours (55%) were designated as amplified. This was decreased to 22 (40%) when the threshold was increased to more than five signals per nucleus. Application of the threshold of at least six signals per nucleus decreased the proportion of amplified samples to 18 (33%).

### Chromogenic *in situ *hybridisation

Two different thresholds of more than five or at least six signals per nucleus were used to establish the optimal cutoff point for HER2 amplification by CISH. When the cutoff of more than five signals per nucleus was applied, 23 tumours (42%) were defined as amplified, and 32 (58%) as NA. In contrast, application of the threshold of at least six signals per nucleus decreased the number of amplified tumours to 20 (36%) and increased the number of NA tumours to 35 (64%).

### Inter-assay agreement

The PathVysion assay kit was chosen as the 'gold standard' for comparisons. Agreement with PathVysion and other techniques used to determine HER2 status is shown in Table 2.

**Table 2:** Concordance between PathVysion and other HER2-testing protocols

Comparison with PathVysion	κ coefficient*	Degree of agreement	Agreement (percentage)	Sensitivity (percentage)	Specificity (percentage)
PharmDx	1.000	Excellent	100	100	100
INFORM (at least six signals per nucleus)	0.916 ± 0.058	Excellent	98	100	94
INFORM (at least five signals per nucleus)	0.799 ± 0.085	Good	91	100	88
INFORM (more than four signals per nucleus)	0.532 ± 0.111	Moderate	76	100	57
CISH (at least six signals per nucleus)	0.876 ± 0.070	Excellent	94	100	94
CISH (at least five signals per nucleus)	0.762 ± 0.091	Good	89	100	88
In-house IHC (positive 3+, negative 0, 1+, 2+)	0.792 ± 0.089	Good	91	89	91
In-house IHC (positive 3+, 2+, negative 0, 1+)	0.475 ± 0.111	Moderate	72	100	57
HercepTest (positive 3+, negative 0, 1+, 2+)	0.645 ± 0.104	Good	83	89	80
HercepTest (positive 3+, 2+, negative 0, 1+)	0.350 ± 0.119	Poor	64	94	49

HER2, human epidermal growth factor receptor 2; CISH, chromogenic *in situ *hybridisation; IHC, immunohistochemistry. (* errors given as ± SD)

There was excellent agreement (100%) between the dual-probe techniques, namely PathVysion and pharmDx. However, agreement between PathVysion and INFORM differed depending on the threshold applied. The lowest level of agreement between the two protocols (76%) occurred with a cutoff of more than four signals per nucleus. This increased to 91% with the threshold of more than five signals per nucleus, and to 98% with a cutoff of at least six signals per nucleus (Table 2).

Similarly, application of different thresholds to CISH altered agreement with PathVysion. An agreement of 89% with a cutoff of more than five signals per nucleus was increased to 94% by changing the cutoff value to at least six signals per nucleus (Table 2).

For IHC, general agreement varied for the HercepTest and in-house IHC protocol depending on whether IHC 2+ samples were classified as positive (grouped with IHC 3+ cases) or negative (grouped with IHC 0/1+ cases) (Table 2). When considering only HER2-negative IHC 0/1+ tumours, both protocols demonstrated 100% concordance with PathVysion (data not shown). Good agreement (89%) was demonstrated between the in-house IHC protocol and PathVysion for the evaluation of IHC 3+ tumours but HercepTest demonstrated poor concordance at 68% (data not shown).

### Details of the unmatched *in situ *hybridisation cases

There were 14 cases that scored differently for HER2 amplification when using the various kits (Table 1). One case scored as amplified with pharmDx but NA with the INFORM and SPoT-Light assays when using all thresholds. The HER2:CEP17 ratio of 2.27 highlighted low amplification but this could not be confirmed with the alternative dual-probe kit (PathVysion) (Table 3). Six cases were scored as amplified (five with low amplification and one with presence of clusters) with the use of the CISH SPoT-Light assay (threshold more than five signals per nucleus) and NA with PathVysion. Four of these tumours had chromosome 17 polysomy, with a mean number of three signals per nucleus for CEP17 with PathVysion. The mean number of CISH HER2 signals per nucleus for the remaining two discrepant cases was 5.6 and clusters. When CISH was used in conjunction with the threshold of at least six signals per nucleus, only one case remained discrepant with the presence of clusters (Table 3).

**Table 3:** Details of the unmatched *in situ *hybridisation cases

Case ID	PathVysion			PharmDx™			INFORM	CISH
	HER2 signals per nucleus	CEP17 signals per nucleus	Ratio	HER2 signals per nucleus	CEP17 signals per nucleus	Ratio	HER2 signals per nucleus	HER2 signals per nucleus
20	5.6	5.3 (P)	1.1	6.2	4.0 (P)	1.6	4.9	6.4
21	2.2	2.5	0.9	5.1	3.3 (P)	1.6	5.6	5.6
22	-	-	-	4.5	2.0	2.3	3.8	3.6
24	2.6	2.5	1.0	3.3	2.6	1.2	4.3	Clusters
25	3.7	3.8 (P)	1.0	6.2	4.0 (P)	1.6	7.7	5.5
26	4.4	2.8	1.6	6.1	4.2 (P)	1.5	5.3	6.6
27	3.4	2.2	1.5	3.8	2.4	1.6	4.8	3.7
28	3.8	3.7 (P)	1.0	4.0	3.4 (P)	1.2	5.7	4.9
29	2.0	2.0	1.0	3.5	2.0	1.7	4.4	3.6
30	4.6	3.3 (P)	1.4	5.7	3.7 (P)	1.5	5.5	3.4
32	2.0	2.5	0.8	2.0	2.0	1.0	4.2	4.9
33	5.6	5.0 (P)	1.1	5.2	3.5 (P)	1.5	4.5	5.7
34	3.3	2.2	1.5	3.3	2.1	1.6	4.1	2.5
35	3.1	3.5 (P)	0.9	2.8	2.7	1.0	4.2	3.3

HER2, human epidermal growth factor receptor 2; CEP17, chromosome enumeration probe 17; P, polysomy; CISH, chromogenic *in situ *hybridisation.

With the INFORM kit and a threshold of more than four signals per nucleus, comparisons with the 'gold standard' PathVysion identified an overvaluation of HER2 amplification in 13 specimens (amplified with INFORM and NA with PathVysion). Overvaluation was not random (*P *= 0.00031, paired-series χ^2 ^test). No false negatives were observed. Chromosome 17 polysomy accounted for six of the unmatched cases considering PathVysion and eight considering PathVysion and pharmDx, but the remaining false positives could not be explained. Application of the following thresholds reduced the number of false positives and improved agreement with PathVysion: more than five signals per nucleus reduced the number of mismatches to five with only one case that could not be explained by a polysomy, and at least six signals per nucleus left only one unmatched case, which could be explained by chromosome 17 polysomy (Table 3). The cutoff points of more than five and at least six signals per nucleus improved agreement from 76% to 91% and 98%, respectively.

### Technical and economic considerations

The minimum time requirements and direct costs for the different techniques are presented in Table 4. CISH was the most time-consuming method because it required immunodetection of the amplification product after hybridisation; however, excluding the in-house kit, it was the cheapest. INFORM was less time-consuming because the staining protocol was automated; PathVysion was the most expensive.

**Table 4:** Summary of protocols and costs

Technical stage	Stage presence or duration				
	
	PathVysion	PharmDx	INFORM	CISH	In-house
Deparaffinisation and rehydration	40 min	33 min	Overnight in the BenchMark	25 min	20 min
Pretreatment	67 min	31 min		21 min	60 min
Digestion	26 min	16 min		16 min	No
Fixation	20 min	No		No	Fixation, blocking, incubation with primary and secondary antibodies and detection, all performed in NexES 90 min
Dehydration	No	6 min		10 min	
Drying	Yes	Yes		Yes	
Denaturation	5 min	5 min		5 min	
Hybridisation	Overnight	Overnight		Overnight	
Washing	4 min	20 min	4 min	11 min	Washing, haematoxylin counterstain (15 sec), dehydration and mounting, 12 min
Dehydration	No	6 min	3 min	No	
Drying	30 min	Fast	Fast	No	
DAPI staining	Yes	Yes	Yes	No	
Detection	No	No	No	147 min	
Total duration	Overnight + 3 h 12 min	Overnight + 1 h 57 min	Overnight + 7 min	Overnight + 3 h 55 min	3 h 2 min
Price (€ per test)	131	85	75	56	4

CISH, chromogenic *in situ *hybridisation; DAPI, 4,6-diamidino-2-phenylindole.

Comparisons of the different *in situ *hybridisation protocols are listed in Table 5. CISH provides several technical advantages over the FISH protocols assessed in this study. First, as CISH uses a chromogenic peroxidase reaction to reveal amplification signals, samples can be assessed with a standard light microscope, allowing simultaneous viewing of staining and histopathology. Second, because the chromogenic labels are stable, samples can be stored easily at ambient temperature for long periods without signal degeneration. However, there are important disadvantages, namely the time associated with immunodetection of the amplification product and the lack of dual chromogenic probes HER2:CEP17, although SPoT-Light chromosome 17 is available.

**Table 5:** Key features of the protocols for comparison

Feature	PathVysion	PharmDx	INFORM	CISH
Reagents	Preparation required	Ready to use	Ready to use	Ready to use
Stability of reagents	pH requires checking	OK	OK	OK
DAPI	Bright	Low intensity	Not provided	-
Background	Orange fluorescence	Strong; green fluorescence	Faint; green fluorescence	-
Stability of signal	Good	Rapid quenching of FITC signal	Good	Excellent
HER2 probe	Spectrum Orange; clear signal but high background fluorescence	Texas red; strong clear signal	FITC; strong clear signal	Chromogenic; peroxidase-based immunodetection, clear signal
CEP17 probe	FITC; very strong signal, with blurring of large signals	FITC; faint and transient	-	-
Tissue morphology	Poor definition; control with H&E to view areas of interest	Very poor definition; control with H&E to view areas of interest	Poor definition; control with H&E to view areas of interest	Good definition, allowing simultaneous analysis of amplification and histopathology
Microscope	Fluorescent	Fluorescent	Fluorescent	Normal light
Storage	1 year at -20°C	1 year at -20°C	1 year at -20°C	Long period at ambient temperature
Threshold	OK	OK	CISH threshold must be applied and borderline cases must be evaluated with a dual-probe method (5–10)	

DAPI, 4,6-diamidino-2-phenylindole; HER2, human epidermal growth factor receptor 2; CEP17, chromosome enumeration probe 17; FITC, fluorescein isothiocyanate; H&E, haematoxylin and eosin staining; CISH, chromogenic *in situ *hybridisation.

Because FISH techniques use fluorophores to localise target DNA, definition of cell morphology is poor; the best is achieved with PathVysion. The key technical disadvantages associated with PathVysion are that the solutions are not ready to use, the reagent pH requires confirmation before use, and fluorescent HER2 probes labelled with Spectrum Orange are hard to detect above the high background fluorescence.

In contrast to the HER2 probe, the pharmDx CEP17 probe was conjugated with fluorescein isothiocyanate (FITC), and positive signals were transient and faint. The high background fluorescence associated with this kit created problems when evaluating the faint CEP17 signals.

The most significant technical disadvantage with INFORM is the lack of dual fluorescence probes HER2:CEP17, which would counter the level of false positives achieved with this kit at its recommended threshold.

## Discussion

Trastuzumab has a proven survival benefit in the treatment of women with HER2-positive breast cancer. Accurate identification of HER2 status is essential for identifying patients who may benefit from HER2-targeted therapy. The protocols currently validated for use in routine diagnosis are IHC for identifying HER2 protein overexpression, and FISH or CISH for determining *HER2 *gene amplification. IHC and FISH are the protocols of choice in the routine diagnosis of HER2 status, providing reliable results. These protocols are reliable only if they are validated within individual laboratories. It is essential that protocols are adhered to, that quality-control and assurance programmes are implemented, and that diagnostic personnel have experience and regular training in score interpretation.

The UK best practice recommends a two-phase testing algorithm based on first-line IHC evaluation and second-line FISH assessment of borderline cases (IHC 2+) [10]. This correlates with recommendations from the College of American Pathologists if the concordance rates are more than 90% between IHC 3+ and FISH amplified, IHC 0 and FISH NA, and 95% between IHC 1+ and FISH NA. The in-house IHC was in accordance with the criteria above; the algorithm was therefore applied to all samples. Our in-house IHC technique has been calibrated with FISH within the framework of the Groupe d'Étude des Facteurs Pronostiques par Immunohistochimie dans le Cancer du Sein (GEFPICS) study [11]. This is not true for HercepTest, for which the agreement was weaker (68%) between IHC 3+ and PathVysion. However, HercepTest has been proposed as the standard IHC method for HER2 status evaluation, capable of overcoming the problems of standardisation and reproducibility between laboratories. In agreement with our results, several studies have demonstrated the excessive sensitivity of this kit [12-14].

The goal of this study was to evaluate the accuracy of four commercial *in situ *hybridisation kits (three FISH and one CISH) and validate a protocol for resolving borderline IHC cases. The PathVysion kit was chosen as the 'gold standard' because it has a proven record of sensitivity and specificity for *HER2 *gene amplification against solid matrix blotting techniques [8].

Concordance between this kit and pharmDx was 100% (the manufacturers report a concordance of 95%); the performance of both kits can therefore be regarded as equivalent. Differences arise in price, time and interpretation. PharmDx is cheaper and faster than PathVysion but interpretation is more difficult (except for highly amplified cases) because of strong background fluorescence and the transient nature of the FITC signal.

Agreement between PathVysion and CISH varied depending on the threshold applied. Excellent agreement (94%) was achieved with a cutoff of at least six signals per nucleus. All patients with amplification of the *HER2 *gene were detected (sensitivity of 100%); however, three false positives (specificity of 94%) were reported with CISH. Two of these were low-level amplifications, having chromosome 17 polysomy and requiring confirmation of diagnosis with one of the dual-probe FISH kits. This threshold recommended by Zymed for the SPoT-Light CISH kit accounted for the occurrence of aneuploid cell populations that can give three to five signals per nucleus, and the manufacturer advised that these be interpreted as NA. In addition, a small proportion of aneuploid cells may contain five to eight signals per nucleus because of DNA replication during the S and G_2_/M phases of the cell cycle and should also be regarded as NA.

Agreement between CISH and FISH in the literature varies between 84% and 100% [15-19]. One study reported 100% agreement between CISH and FISH when samples scoring more than two signals per nucleus with HER2 CISH were systematically controlled for by using a chromosome 17 CISH probe on adjacent sections [19]. CISH provides a good alternative to FISH in laboratories that lack access to fluorescence microscopy. However, we recommend that for borderline cases with CISH scores of more than five to ten signals per nucleus, diagnosis must be confirmed by using either a dual-probe FISH kit as used in this study, or an internal control such as the chromosome 17 SPoT-Light probe for CISH.

With the manufacturer's recommended threshold of at least four signals per nucleus, concordance between PathVysion and INFORM was only 76%, with a sensitivity of 100% but a specificity of 57% (13 false positives). Application of the thresholds of more than five or at least six signals per nucleus to the INFORM results increased concordance with PathVysion to 91% and 98%, respectively. The small amount of discordance was due to CEP17 polysomy, except in one unexplained case. As with CISH, we advise a control with a dual-probe FISH assay for borderline INFORM cases (five to ten signals per nucleus). Our results compare well with those recently published on an evaluation of three scoring methods for FISH: dual-probe HER2:CEP17 or single-probe HER2 assay with a threshold of more than four or more than six signals per nucleus [20]. This study also recommended a threshold of more than six signals per nucleus for FISH or a dual probe HER2:CEP17 FISH assay for optimal HER2 scoring. This was based on high concordance rates with IHC or mRNA/assay/nucleic acid sequence-based amplification and are in agreement with the recent American Society of Clinical Oncology/College of American Pathologists guidelines for HER2 testing [21]. The theoretical advantage of dual-probe FISH analysis is the ability to distinguish chromosomal aneuploidy from amplification by using a differentially labelled reference probe (CEP17). Although a single probe-based assay, the main benefit of INFORM over other assays is that it is an automated system.

## Conclusion

**Figure 1:**
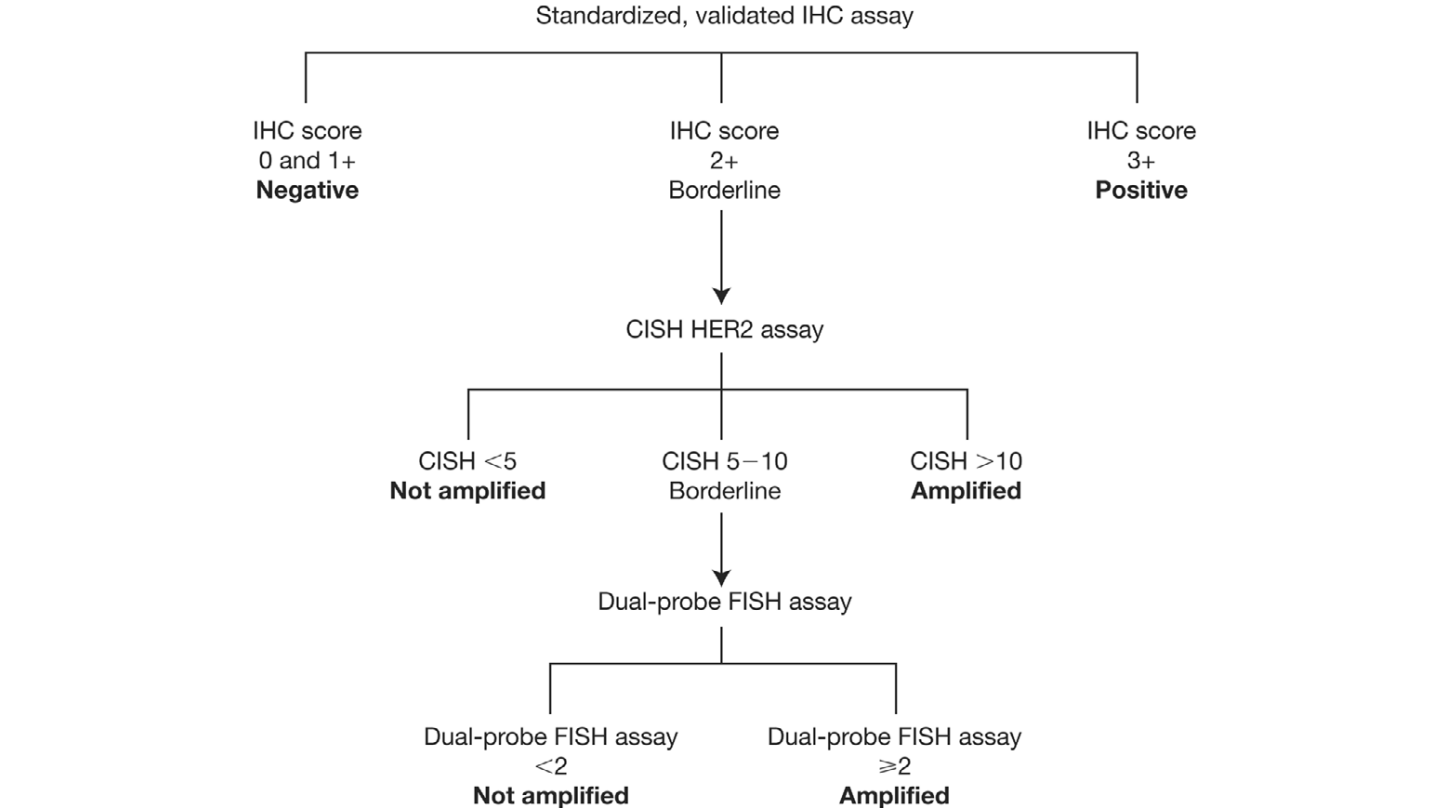
Future algorithm for testing for human epidermal growth factor receptor 2 (HER2). IHC, immunohistochemistry; CISH, chromogenic *in situ *hybridisation; FISH, fluorescence *in situ *hybridisation.

We can draw several conclusions from this study. First, we can validate CISH as a promising alternative to FISH for determining gene amplification, because it is easy to interpret and the equipment can be readily found in most pathology departments. The potential of CISH as a HER2 status assay for routine practice has been the subject of an international multicentre ring study, to which we have contributed, to validate CISH against the current 'gold standards' [22]. Second, the automated mono-probe assay INFORM provides the opportunity for a more rapid evaluation of samples compared with CISH and the two dual-probe FISH assays evaluated in this study, provided that an appropriate threshold is applied. Despite the clear benefits attributed to these two approaches, we would advise that borderline CISH or single-probe FISH results are controlled for by using a dual-probe FISH assay. The ideal situation for pathologists in the future would be for FDA approval of CISH in routine diagnosis and for automation of the assay. The algorithm currently employed would then change to that presented in Figure 1.

## Abbreviations

CEP17 = chromosome enumeration probe 17; CISH = chromogenic *in situ *hybridisation; DAPI = 4,6-diamidino-2-phenylindole; FDA = Food and Drug Administration; FISH = fluorescence *in situ *hybridisation; FITC = fluorescein isothiocyanate; HER2 = human epidermal growth factor receptor 2; IHC = immunohistochemistry; NA = non-amplified.

## Competing interests

The authors declare that they have no competing interests.

## Authors' contributions

AC and FP-L participated in the design of the study, performed the blind scoring of CISH and FISH samples, and the interpretation and statistical analysis of the results, and wrote the manuscript. FM and NL performed blind scoring of the IHC samples. All authors read and approved the final manuscript.
